# Repair of left brachial artery aneurysm with excision and interposition grafting: a case report

**DOI:** 10.1093/jscr/rjag536

**Published:** 2026-06-29

**Authors:** Mark Ragheb, Alexandra F Shapiro, Matthew Samra

**Affiliations:** St. George’s University School of Medicine, St. George’s GD-105, Grenada; Department of General Surgery, Jersey Shore University Medical Center, Neptune, NJ 07753, United States; Department of Vascular Surgery, Jersey Shore University Medical Center, Neptune, NJ 07753, United States

**Keywords:** brachial artery aneurysm, peripheral artery aneurysm, interposition vein graft, myoclonus dystonia

## Abstract

True brachial artery aneurysms are uncommon and most commonly associated with trauma or connective tissue disorders, with thromboembolism. We report a 41-year-old male with myoclonus dystonia presenting with a 4-week history of an enlarging left arm mass. Imaging revealed a 1.3 cm saccular brachial artery aneurysm, likely due to repetitive blunt trauma. The patient underwent successful excision with reversed basilic vein interposition grafting and was discharged without complications. This case highlights the role of repetitive minor trauma in aneurysm formation and the importance of early surgical management to prevent ischemic complications.

## Introduction

Peripheral artery aneurysms are uncommon vascular pathologies characterized by localized dilation of an artery, most frequently affecting the popliteal and femoral arteries. Upper extremity aneurysms are particularly rare, with brachial artery aneurysms representing about 0.5% of all peripheral artery aneurysms and true brachial artery aneurysms presenting with an incidence of 0.17% [1]. These aneurysms may arise secondary to trauma, iatrogenic injury, atherosclerosis, or connective tissue disorders such as Ehlers-Danlos Syndrome.

Unlike abdominal aortic aneurysms where rupture is the primary concern, peripheral aneurysms in the upper extremity pose their greatest risk through thromboembolic complications rather than rupture. This can lead to digital ischemia, gangrene, and limb-threatening ischemia.

Due to the rarity of true brachial artery aneurysms and the paucity of evidence guiding their management, individual case reports remain valuable contributions to the literature. In this report, we present a case of a left brachial artery aneurysm of unknown etiology repaired by excision and interposition grafting using the left basilic vein.

## Case presentation

A 41-year-old male with a history of factor VIII, anxiety, myoclonus dystonia presented to the emergency department 2 weeks ago with a 4-week history of an enlarging mass in the left upper arm. He denied chest pain, shortness of breath, fevers, chills, nausea, vomiting, back pain, or abdominal pain, and the remainder of the review of systems was unremarkable. Ultrasound evaluation at that time confirmed a left brachial artery aneurysm, and the patient was subsequently referred to vascular surgery for further management.

Computed tomography (CT) of the left upper extremity confirmed the presence of a saccular aneurysm of the left brachial artery at the level of the mid humeral diaphysis measuring 1.32 cm in diameter (Fig. 1). The patient denied a known history of trauma to the area, however stated that due to his myoclonus dystonia he does sometimes hit his body uncontrollably against things, suggesting blunt trauma as a likely cause. He endorsed mild soreness in the left arm, accompanied by intermittent numbness and tingling in the hands and fingertips. Following discussion of the risks and benefits of surgery, the patient elected to proceed with elective surgical repair after obtaining cardiac clearance.

**Figure 1:**
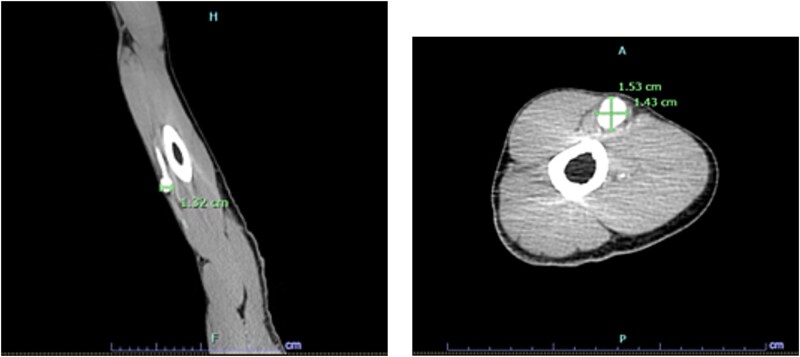
Sagittal CT (right) and axial (left) revealing a saccular aneurysm arising from the brachial artery is seen at the level of the mid humeral diaphysis.

The patient underwent excision of a left brachial artery aneurysm with interposition grafting. An incision was made along the course of the basilic vein, which was carefully dissected and harvested from the elbow to the axillary vein obtaining an ~10 cm segment for use as a conduit. The brachial artery was then exposed and noted to be densely adherent to the median nerve, requiring meticulous dissection to avoid injury. Proximal and distal control of the artery was obtained with vessel loops, and systemic heparinization was administered. The aneurysmal segment was excised, resulting in a 4.5–5 cm arterial defect. A reversed basilic vein graft was then used for interposition, with proximal and distal end-to-end anastomoses performed using 6-0 Prolene sutures. After restoration of flow, there was excellent distal perfusion with a strong palpable radial pulse. Hemostasis was achieved, and the wound was irrigated and closed in layers. The patient tolerated the procedure well and was discharged on postoperative day one after routine observation (Figs 2 and 3).

**Figure 2:**
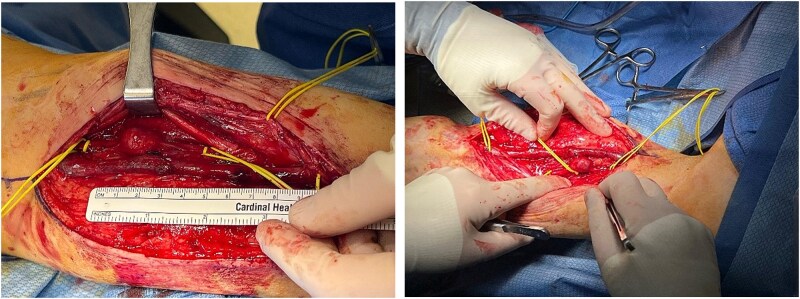
Intraoperative view of the dissected left arm demonstrating the brachial artery with a large 1.3 cm aneurysm. The basilic vein is visible, and the ulnar and median nerves were carefully isolated.

**Figure 3:**
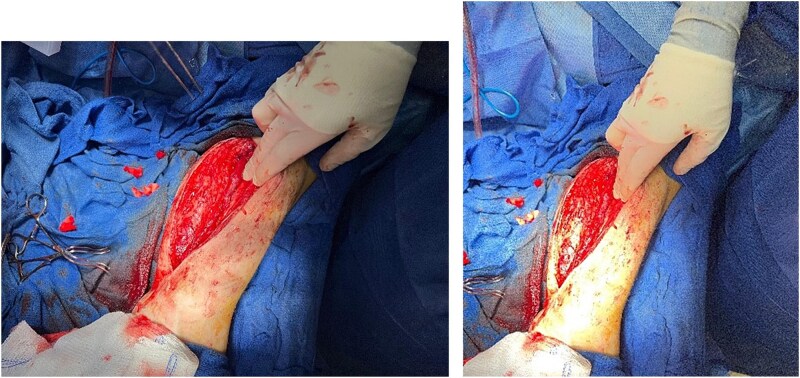
Post-excision reconstruction showing the affected arterial segment removed and interposition grafting with reversed autologous basilic vein.

## Discussion

Trauma is the most common cause of brachial artery aneurysms, particularly in younger individuals, and includes iatrogenic causes as well as blunt or penetrating trauma. In one study of upper extremity aneurysms, traumatic mechanisms accounted for ~41.2% of cases with the brachial artery being the most commonly affected site [2]. In this case, the patient’s history of myoclonus dystonia likely contributed to arterial injury and aneurysm formation despite the absence of a single identifiable traumatic event.

The pathophysiology of post-traumatic true aneurysm formation involves partial disruption of the arterial wall. Unlike pseudoaneurysms, which result from full-thickness arterial wall injury with containment by surrounding tissue, true aneurysms arise from intimal injury with preservation and progressive dilation of the media and adventitia [3]. Key features include intimal disruption and fragmentation of the internal elastic lamina [4]. This partial wall injury initiates a chronic degenerative process that can lead to gradual aneurysmal dilation over time. Clinically, traumatic aneurysms may present acutely or develop insidiously over years, highlighting the importance of maintaining suspicion in patients with repeated or minor trauma [5]. In our patient, the aneurysm presented as an enlarging mass over 4 weeks, consistent a subacute presentation.

Definitive management is surgical, and prevention of recurrence relies on both surgical and medical strategies. Complete excision with adequate margins is critical to avoid leaving behind diseased arterial tissue [6]. Autologous vein interposition grafts such as the basilic vein are generally preferred over prosthetic conduits due to superior long-term patency and lower complication rates [7]. Postoperative surveillance, including duplex ultrasonography and clinical follow-up, is recommended to detect graft stenosis early and ensure continued arterial patency [8]. Lifelong antithrombotic therapy, coupled with management of cardiovascular risk factors such as smoking, hypertension, and dyslipidemia, reduces the risk of recurrence [8, 9]. In younger patients with aneurysms following minor trauma, evaluation for connective tissue disorders should be considered since these patients may be predisposed to recurrent or multifocal vascular lesions [10].

Long-term complications such as para-anastomotic aneurysm formation, which can occur years after reconstruction, highlights the importance of continued surveillance [10]. This case illustrates that subtle, repetitive trauma can result in true brachial artery aneurysms, and prompt recognition with appropriate surgical management can lead to excellent outcomes. Clinicians should maintain an index of suspicion in patients with risk factors for repeated trauma or involuntary movements, as early intervention can prevent complications such as distal embolization, thrombosis, or rupture.
